# Reliability and Validity of the KIPPPI: An Early Detection Tool for Psychosocial Problems in Toddlers

**DOI:** 10.1371/journal.pone.0049633

**Published:** 2012-11-21

**Authors:** Ingrid Kruizinga, Wilma Jansen, Carolien L. de Haan, Hein Raat

**Affiliations:** 1 Department of Public Health, Erasmus University Medical Center, Rotterdam, The Netherlands; 2 Department of Youth Policy, Rotterdam Municipal Health Service (GGD Rotterdam-Rijnmond), Rotterdam, The Netherlands; The University of Queensland, Australia

## Abstract

**Background:**

The KIPPPI *(*Brief Instrument Psychological and Pedagogical Problem Inventory**)** is a Dutch questionnaire that measures psychosocial and pedagogical problems in 2-year olds and consists of a KIPPPI Total score, Wellbeing scale, Competence scale, and Autonomy scale. This study examined the reliability, validity, screening accuracy and clinical application of the KIPPPI.

**Methods:**

Parents of 5959 2-year-old children in the Rotterdam area, the Netherlands, were invited to participate in the study. Parents of 3164 children (53.1% of all invited parents) completed the questionnaire. The internal consistency was evaluated and in subsamples the test-retest reliability and concurrent validity with regard to the Child Behavioral Checklist (CBCL). Discriminative validity was evaluated by comparing scores of parents who worried about their child’s upbringing and parent’s that did not. Screening accuracy of the KIPPPI was evaluated against the CBCL by calculating the Receiver Operating Characteristic (ROC) curves. The clinical application was evaluated by the relation between KIPPPI scores and the clinical decision made by the child health professionals.

**Results:**

Psychometric properties of the KIPPPI Total score, Wellbeing scale, Competence scale and Autonomy scale were respectively: Cronbach’s alphas: 0.88, 0.86, 0.83, 0.58. Test-retest correlations: 0.80, 0.76, 0.73, 0.60. Concurrent validity was as hypothesised. The KIPPPI was able to discriminate between parents that worried about their child and parents that did not. Screening accuracy was high (>0.90) for the KIPPPI Total score and for the Wellbeing scale. The KIPPPI scale scores and clinical decision of the child health professional were related (p<0.05), indicating a good clinical application.

**Conclusion:**

The results in this large-scale study of a diverse general population sample support the reliability, validity and clinical application of the KIPPPI Total score, Wellbeing scale and Competence scale. Also, the screening accuracy of the KIPPPI Total score and Wellbeing scale were supported. The Autonomy scale needs further study.

## Introduction

The importance of early detection of psychosocial problems, such as social-emotional and behavioural problems in toddlers is increasingly recognized. [1], [2], [3] In the Netherlands, in approximately 8–9 percent of preschool children, child health professionals identify psychosocial problems. [4], [5] Psychosocial problems are associated with psychological disorders later in life. [6], [7] Therefore, it is important to detect and treat psychosocial problems at a young age, because early detection and treatment may contribute to a reduction of problems and an increase in competencies at an older age. [8], [9] However, studies show that a relatively small number of children with psychosocial problems are identified by child health professionals (i.e. 29% of the children who scored in the clinical range of the CBCL Total Problem score) [5] and are being referred to mental health services (i.e. 13% of the children who scored in the clinical range of the CBCL Total Problem score). [10] The accuracy of identification of psychosocial problems should be enhanced. [11] To facilitate early detection of psychosocial problems in toddlers, child health professionals can use reliable and valid parent-completed questionnaires. [12], [13], [14].

The toddler KIPPPI [15] (KIPPPI is a Dutch acronym for Brief Instrument Psychological and Pedagogical Problem Inventory) was developed in the Netherlands and measures psychosocial problems in 2-year olds, which might be possible pedagogical challenges for the parents. The KIPPPI has 67 items and consist of a Wellbeing scale (31 items), Competence scale (25 items) and an Autonomy scale (11 items). The KIPPPI Total score is the sum of the scale scores. Child health professionals use the KIPPPI as an early detection tool during well-child visits to assess the child’s psychosocial problems that might also be related to pedagogical problems. The KIPPPI is specifically developed for use in the preventive child health care and is widely used in the Netherlands. As many aspects of psychosocial problems are addressed in the questionnaire, the KIPPPI can be used by the child health professional to guide conversation with the parent.

Little is known about the reliability and validity of the KIPPPI. The objective of this study was to determine in a large general population sample of 2-year old children: the score distribution of the KIPPPI (mean KIPPPI scores and standard deviations for the total population as well as for subgroups by child’s gender and ethnicity; and floor and ceiling effects) and the following psychometric properties of the KIPPPI; the reliability of the KIPPPI scale scores (internal consistency and test-retest reliability); the validity of the KIPPPI scale interpretation (concurrent validity and discriminative validity); the screening accuracy of the KIPPPI was evaluated relative to the Child Behavioral Checklist 1.5–5 (CBCL1.5-5), a well-validated questionnaire that measures behavioural, emotional and social problems in preschool children. Additionally we evaluated the clinical application; whether the KIPPPI scores were related to the clinical decision of the child health professionals.

## Methods

### Ethics Statement

Part of the data became available in the context of the government-approved routine health examinations of the preventive child health care. Anonymous data were used in this study and the questionnaires were completed on a voluntary basis. Parents received written information on the study and were free to object to participation. Observational research does not fall within the ambit of the Dutch Act on research involving human subjects and does not require the approval of an ethics review board. Written informed consent was obtained from a subgroup of parents that participated in a substudy to evaluate the test-retest reliability and to compare KIPPPI scores with CBCL1.5-5 scores, because these data were not anonymous and were not collected as part of the routine health examinations. The study was conducted in accordance with the WMA Declaration of Helsinki principles. We received a formal waiver (i.e. declaration of no objection) from The Medical Ethics Committee of the Erasmus Medical Center Rotterdam.

### Data Collection

The present study was conducted among two-year-old children and their parents, who were invited between April 2010 and April 2011 by child health care organizations in the larger Rotterdam area, the Netherlands, for well-child visits: A few weeks before the well-child visit was scheduled, parents of 5959 children were sent a child health monitor questionnaire by mail, including among others the KIPPPI and CBCL1.5-5, and written information about the study. Parents decided for themselves whether the father or mother would complete the questionnaire. Parents handed in the completed child health monitor questionnaire at the well child visit. The child health professional used the parent-completed KIPPPI as both a guide for the conversation with parents and as a tool for early detection of psychological and pedagogical problems. Based on the conversation with the parent and the completed child health monitor questionnaire, the child health professional made a clinical decision whether a child is to be referred to a mental health care professional (e.g. psychologist) or whether a follow-up consultation is required. The child health professional registered the clinical decision on a separate registration form or in a digital medical record system. Although the KIPPPI can be scored, in this study the child health care professionals did not calculate scores since cutpoints were not empirically determined at the time.

Parents of 3655 (61.3%) children attended the well-child visit. The remaining parents (38.7%) neither attended the well-child visit nor completed the child health monitor questionnaire. Of those parents that did attend the well-child visit, 3164 (86.6%) had completed the child health monitor questionnaire. Children were excluded from the analyses if they were under treatment by a mental health professional at the time of inclusion (n = 1; 0.03%), or if the KIPPPI contained more than 25% missing items on the KIPPPI scales (n = 431, 13.6%). After exclusion, a study population of 2732 (86.3%) children was eligible for this study. The CBCL1.5-5 [16] was also included in the child health monitor questionnaire but only for research purposes (i.e. evaluating the concurrent validity and screening accuracy of the KIPPPI). Parents of 2016 (55.2%) children, who attended the well-child visit, also completed the CBCL1.5-5 in addition to the KIPPPI.

After the well-child visit, 225 parents were sent another copy of the KIPPPI to assess the test-retest reliability of the KIPPPI. The parents of 90 (40.0%) children returned the KIPPPI. The range of the period between completion of questionnaires was 5–78 days (*mean* = 38.6, *SD* = 17.5).

Mean child age was 24.1 months (*SD* = 1.2), 47.7% were girls, and 72.1% of the children had a native Dutch ethnic background. [17] Mean age of the mother was 33.5 years (*SD* = 4.8) and mean age of the father was 36.2 years (*SD* = 5.5). In 92.6% of the cases, the mother or both parents were the respondent(s). See Table 1 for information on demographic characteristics of the study population.

**Table 1:** Characteristics of the study population, N = 2732.

	% of participants	Mean (SD)
**Characteristics Mother**		
Age (years)		33.5 (4.8)
Born in the Netherlands	77.9	
**Characteristics Father**		
Age (years)		36.2 (5.5)
Born in the Netherlands	76.6	
**Characteristics Children**		
Age (months)		24.1 (1.2)
Gender (girls)	47.7	
Ethnic background (Dutch)^1^	72.1	
**Family characteristics**		
Two-parent household	88.6	
One-child family	40.8	
Respondent (mother or both parents)	92.6	

1 A child is considered Dutch when both parents were born in the Netherlands.

### Measures

The KIPPPI has 67 items regarding psychosocial child problems, which might be possible pedagogical challenges for the parents. The child health professional discusses the items with high scores (indicating a problem) with parents and assesses whether the difficulties stem from a problem in the child (i.e. psychosocial), or the parent (pedagogical), or the parent-child interaction. The KIPPPI consist of a Wellbeing scale (31 items), Competence scale (25 items) and an Autonomy scale (11 items). The response options range from 0 (‘(almost) never’) to 3 (‘(almost) always’), or reversed if the item is positively formulated. The KIPPPI Total score is the sum of the scale scores. Responses were summed for each scale and missing values on the KIPPPI items were imputed with within scale person-means. [18] High scores on the KIPPPI are less favourable. The possible score range of the KIPPPI Total score is 0–201, of the Wellbeing scale is 0–93, of the Competence scale 0–75, of the Autonomy scale 0–33. Wellbeing consists of five subscales that measure difficulties of the child with eating/drinking (4 items) and sleeping (3 items) and whether the child shows problems with activity (5 items), mood (5 items) and behaviour (14 items). The Competence scale consists of four subscales that measure unfavourable child cognitive development (4 items) and whether the child shows problems with language (4 items), play (3 items) and contact (14 items). The Autonomy scale consists of three subscales and measure whether the child has problems with toilet training (4 items), motor skills (3 items) and independence (4 items). See Table 2 for an overview of the (sub)scales and item examples. Additionally, the KIPPPI contains six additional items regarding the child’s physical health, each with three response options (‘good’, ‘average, ‘bad’/‘never’, ‘sometimes’, ‘often’), with a possible score range of 0–12. The physical health scale does not add to the KIPPPI Total score.

**Table 2:** Overview of the (sub)scales of the KIPPPI and item examples^1^ with response options.

Scale	Subscale	Item example	Response options
Wellbeing			
	eating/drinking	My toddler does not like certain food or drinks	(almost) never, sometimes, often, (almost) always
	sleeping	My toddler has nightmares	idem
	activity	My toddler is overactive	idem
	mood	My toddler is nervous, tense	idem
	behaviour	My toddler is bad tempered	idem
Competence			
	cognitive development	My toddler is easily persuaded to start a new activity	(almost) always, often, sometimes, (almost) never
	language	My toddler speaks in sentences of 2 words or more	idem
	play	My toddler likes playing games (e.g. peekaboo)	idem
	contact	My toddler has difficulty adjusting	(almost) never, sometimes, often, (almost) always
Autonomy			
	toilet training	My toddler wets his/her pants or diaper	(almost) never, sometimes, often, (almost) always
	motor skills	My toddler bumps into things or falls	idem
	indepence	My toddler tries to repair something that is broken	(almost) always, often, sometimes, (almost) never

1 The KIPPPI is a Dutch questionnaire and for the purpose of this article some items are translated.

In addition to the KIPPPI, parents completed the CBCL1.5-5 in order to evaluate the concurrent validity and the screening accuracy of the KIPPPI. The well-validated [16] 100-item CBCL1.5-5 is designed for children aged 18-months to 5-years and has two domains (Internalising and Externalising) and provides a Total Problem score. Answers are given on a 3-point scale (‘not true’, ‘somewhat or sometimes true’ and ‘very true or often true’).

### Analyses

#### Score distribution

Score distribution was evaluated by assessing the mean scale scores and standard deviations, and the presence of floor and ceiling effects (i.e. >15% of the respondents have the minimal and/or maximal score). [19] Independent t-tests were performed to test the differences in mean KIPPPI scores between subgroups for child gender and ethnicity.

#### Reliability

Cronbach’s alpha was used to evaluate the internal consistency of the KIPPPI-Total score, Wellbeing, Competence and Autonomy scales and their subscales. An alpha of 0.70 or higher was considered acceptable. [20] Test-retest reliability of the KIPPPI-scales was assessed with the Intraclass Correlation Coefficients (ICC), using a two-way random effect model with absolute agreement. An ICC of 0.70 or higher is considered to indicate acceptable test-retest reliability. [19].

#### Validity

Concurrent validity was evaluated by assessing the Pearson correlation coefficients between KIPPPI and CBCL1.5-5 scale scores. Concurrent validity is hypothesised to be expressed in: large positive correlations between (a) KIPPPI-Total score, (b) KIPPPI Wellbeing and CBCL1.5-5 Internalising, Externalising and Total Problem scores, since the content of the items of the KIPPPI Total score and KIPPPI Wellbeing scale most resemble the items of the CBCL1.5-5. Furthermore we hypothesised there would be small to medium positive correlations between (c) KIPPPI Competence scale, (d) KIPPPI Autonomy scale and CBCL1.5-5 Internalising, Externalising and Total Problem scores, because the content of the items of the KIPPPI Competence scale and KIPPPI Autonomy scale have less overlap with items on the CBCL1.5-5. A correlation of 0.10 is considered small, 0.30 is considered medium and >0.50 is considered large. [21].

Discriminative validity of the KIPPPI was evaluated by the ability of the KIPPPI to discriminate between a subgroup of parents who did and did not report being worried about their child’s upbringing. We hypothesised that discriminative validity will be reflected in less favourable KIPPPI scores for children of parents who are worried about their child. [22] Regression analyses were used to evaluate the relationship between parental worry as independent variable and KIPPPI (scale) scores as dependent variable, corrected for confounding effects of child’s gender and ethnicity. We hypothesised that parental worry is a significant predictor of KIPPPI scores. Effect sizes were defined as *Cohen’s d = [mean(worried)–mean(not worried)]/SD_worried_*_;_ [21] 0.20≤*d*<0.50 indicates a small effect, 0.50≤*d*<0.80 indicates a medium effect and *d*≥0.80 indicates a large effect.

**Table 3:** Score distributions, internal consistency and test-retest reliability of the KIPPPI-scales, N_total_ = 2732.

KIPPPI scale (# items)	Mean (SD)Total	Mean (SD)Boys (N = 1409)	Mean (SD)Girls (N = 1304)	Mean (SD)Native (N = 1969)	Mean (SD)Non-native (N = 763)	%min^1^Total	%max^1^Total	Cronbach’sα Total	Test-retest ICC^2^N = 90
KIPPPI TOTAL (67)	41.7 (14.5)	43.6^a^ (14.7)	39.6^a^ (14.1)	40.5^b^ (14.0)	44.8^b^ (15.5)	0.0	0.0	0.88	0.80
Physic. Health (6)	1.0 (1.2)	1.1 (1.3)	1.0 (1.2)	1.0 (1.2)	1.1 (1.3)	43.7	0.0	0.38	0.87
Wellbeing (31)	17.0 (8.4)	17.6^a^ (8.6)	16.3^a^ (8.2)	16.4^b^ (8.2)	18.6^b^ (8.7)	0.0	0.0	0.86	0.76
Eating/Drinking (4)	2.4 (2.0)	2.4 (2.0)	2.3 (2.0)	2.3 (1.9)	2.4 (2.1)	21.3	0.0	0.70	0.63
Sleeping (3)	1.3 (1.4)	1.3 (1.4)	1.3 (1.4)	1.3^b^ (1.4)	1.5^b^ (1.4)	35.7	0.0	0.59	0.82
Activity (5)	3.5 (2.3)	3.7^a^ (2.4)	3.2^a^ (2.2)	3.4^b^ (2.4)	3.6^b^ (2.3)	9.3	0.0	0.67	0.78
Mood (5)	1.1 (1.6)	1.1 (1.5)	1.2 (1.7)	0.9^b^ (1.3)	1.7^b^ (1.9)	46.0	0.0	0.60	0.45
Behaviour (14)	8.7 (4.7)	9.0^a^ (4.8)	8.3^a^ (4.5)	8.4^b^ (4.6)	9.3^b^ (4.7)	2.0	0.0	0.82	0.80
Competence (25)	11.6 (7.1)	12.2^a^ (7.3)	11.0^a^ (6.8)	11.0^b^ (6.7)	13.1^b^ (7.6)	1.1	0.0	0.83	0.73
Cognitive development (4)	2.0 (1.5)	2.0 (1.5)	1.9 (1.5)	1.9^b^ (1.4)	2.1^b^ (1.6)	13.5	0.0	0.61	0.46
Language (4)	2.6 (2.8)	3.1^a^ (3.0)	2. 1^a^ (2.4)	2.3^b^ (2.6)	3.4^b^ (3.0)	30.4	0.8	0.79	0.79
Play (3)	0.8 (1.1)	0.8^a^ (1.2)	0.7^a^ (1.1)	0.7^b^ (1.0)	0.9^b^ (1.2)	56.2	0.0	0.60	0.62
Contact (14)	5.8 (4.5)	5.8 (4.5)	5.8 (4.5)	5.6^b^ (4.3)	6.3^b^ (4.8)	8.8	0.0	0.82	0.71
Autonomy (11)	13.1 (3.3)	13.8^a^ (3.1)	12.4^a^ (3.4)	13.2 (3.1)	13.0 (3.6)	0.0	0.0	0.58	0.60
Toilet training (4)	7.9 (2.0)	8.3^a^ (1.8)	7.5^a^ (2.2)	8.0^b^ (1.9)	7.7^b^ (2.4)	1.1	1.8	0.59	0.60
Motor skills (3)	1.1 (0.8)	1.2^a^ (0.8)	1.1^a^ (0.8)	1.1 (0.8)	1.1 (0.9)	17.7	0.0	0.18	0.70
Independence (4)	4.1 (1.9)	4.3^a^ (1.9)	3.8^a^ (1.9)	4.0^b^ (1.8)	4.2^b^ (2.0)	0.0	0.0	0.49	0.74

1 % of respondents with the lowest (min) and highest (max) BITSEA scale score (ceiling/floor).

2 Test-retest Intraclass Correlation Coefficients are all significant, *p*<0.01.

a = significant difference in mean BITSEA scores between boys and girls, *p*<0.05.

b = significant difference in mean BITSEA scores between native and non-native children, *p*<0.05.

#### Screening accuracy

Screening accuracy for the KIPPPI Total scores and scores on Wellbeing, Competence and Autonomy scales was evaluated against the CBCL1.5-5 as a golden standard (i.e. Total Problem score in the clinical range), by calculating the Receiver Operating Characteristic (ROC) curves. The ROC curve is a plot of sensitivity as a function of 1-specificity for all possible cutpoints. The greater the area under the curve (AUC), the more discriminative the KIPPPI scores are. An AUC greater than 0.90 indicates high accuracy, AUC of 0.70–0.90 indicates moderate accuracy, 0.50–0.70 low accuracy, and 0.50 chance level accuracy [23].

A method to determine the optimal cutpoint for a test is calculating the Youden’s index, which is defined as the maximum vertical distance between the ROC curve and the diagonal or chance line and is calculated as *Youden’s index = sensitivity+specificity-1*. Screening accuracy for various cutpoints was evaluated by sensitivity, specificity, positive and negative likelihood ratio’s (LHR^+^ and LHR^−^) and diagnostic odds ratio (OR).

**Table 4:** Concurrent validity (Pearson correlation coefficients) between KIPPPI scales and CBCL1.5-5 Internalising, Externalising and Total Problem score, N = 2016.

	CBCL scales		
	Internalising	Externalising	Total Probem
**KIPPPI scales**			
KIPPPI TOTAL	0.60	0.63	0.68
Physical health	0.28	0.20	0.27
Wellbeing	0.55	0.74	0.72
Eating/Drinking	0.26	0.21	0.26
Sleeping	0.27	0.26	0.37
Activity	0.31	0.61	0.51
Mood	0.49	0.36	0.46
Behaviour	0.49	0.73	0.67
Competence	0.48	0.32	0.43
Cognitive development	0.23	0.17	0.22
Language	0.18	0.17	0.19
Play	0.28	0.33	0.33
Contact	0.50	0.26	0.40
Autonomy	0.18	0.23	0.23
Toilet training	0.04	0.10	0.07
Motor skills	0.23	0.32	0.33
Independence	0.16	0.15	0.17

Note: Underlined correlation is non-significant (*p*>0.05), all other correlations are significant, *p*<0.01.

Sensitivity is the proportion of true positives that are correctly identified by the test; specificity is the proportion of true negatives that are correctly identified by the test. In clinical practice, however, the test result is all that is known, knowledge whether or not someone is correctly classified is not available.

To overcome this problem, likelihood ratio’s can be calculated. LHR^+^ is the ratio of the probability of a positive test result if the outcome is positive (true positive) to the probability of a positive test result if the outcome is negative (false positive); LHR^+^ = (*sensitvitiy/1−specificity*). LHR^−^ is the ratio of the probability of a negative test result if the outcome is positive (false negative) to the probability of a negative test result if the outcome is negative (true negative); LHR^−^ = (*1−sensitivity/specificity*). Tests with high screening accuracy have LHR^+^ greater than 7 and LHR^−^ smaller than 0.30 [24].

**Table 5:** Discriminative ability of the KIPPPI between subgroups differing in parental worries about the child’s upbringing.

	Parental worries			
	Mean (SD)		Beta^1^	Effect size^2^
	Not worried N = 2109	Worried N = 604		
KIPPPI TOTAL	39.0 (13.1)	51.2 (15.0)	11.87	0.81^a^
Physical health	0.9 (1.1)	1.4 (1.4)	0.49	0.36^c^
Wellbeing	15.3 (7.4)	22.8 (8.8)	7.38	0.85^a^
Eating/drinking	2.1 (1.9)	3.1 (2.1)	0.94	0.48^c^
Sleeping	1.2 (1.3)	1.8 (1.6)	0.62	0.38^c^
Activity	3.2 (2.2)	4.5 (2.6)	1.28	0.50^b^
Mood	1.0 (1.4)	1.7 (1.9)	0.69	0.37^c^
Behaviour	7.8 (4.1)	11.7 (5.1)	3.84	0.76^b^
Competence	10.8 (6.6)	14.4 (7.8)	3.40	0.46^c^
Cognitive development	1.9 (1.4)	2.3 (1.6)	0.39	0.25^c^
Language	2.4 (2.6)	3.3 (3.1)	0.78	0.29^c^
Play	0.7 (1.0)	1.1 (1.3)	0.46	0.31^c^
Contact	5.4 (4.3)	7.2 (4.8)	1.78	0.38^c^
Autonomy	12.9 (3.2)	14.0 (3.3)	1.10	0.33^c^
Toilet training	7.8 (2.0)	8.2 (2.1)	0.33	0.19^c^
Motor skills	1.1 (0.8)	1.4 (0.9)	0.34	0.33^c^
Independence	4.0 (1.9)	4.4 (1.9)	0.42	0.21^c^

1 Unstandardized Beta’s are corrected for confounding effects of child’s gender and ethnicity and significant, p<0.01.

2 Difference of the means divided by SD in the subgroup ‘worried’.

a indicates a large effect (*d*≥0.80).

b indicates a medium effect (0.50≤ *d* <0.80).

c indicates a small effect (0.20≤ *d* <0.50).

The diagnostic odds ratio of a test is the ratio of the odds of a positive test result when having the ‘disorder’ relative to the odds of a positive test result when not having the ‘disorder’ and can be calculated as (sensitivity*specificity)/((1−sensitivity)*(1−specificity)) = LHR^+^/LHR ^−^. The values of OR ranges from zero to infinity, with higher values indicating better discriminatory test performance. Potentially useful tests tend to have diagnostic odds ratios well above 20. [24] A value of 1 means that a test does not discriminate between people with and people without the ‘disorder’. Values lower than 1 indicate improper test interpretation, meaning more negative tests among the people with the ‘disorder’. [25] AUC, Youden’s index, sensitivity, specificity, LHR^+^, LHR^−^ and OR are independent of the prevalence of the ‘disorder’.

**Figure 1:**
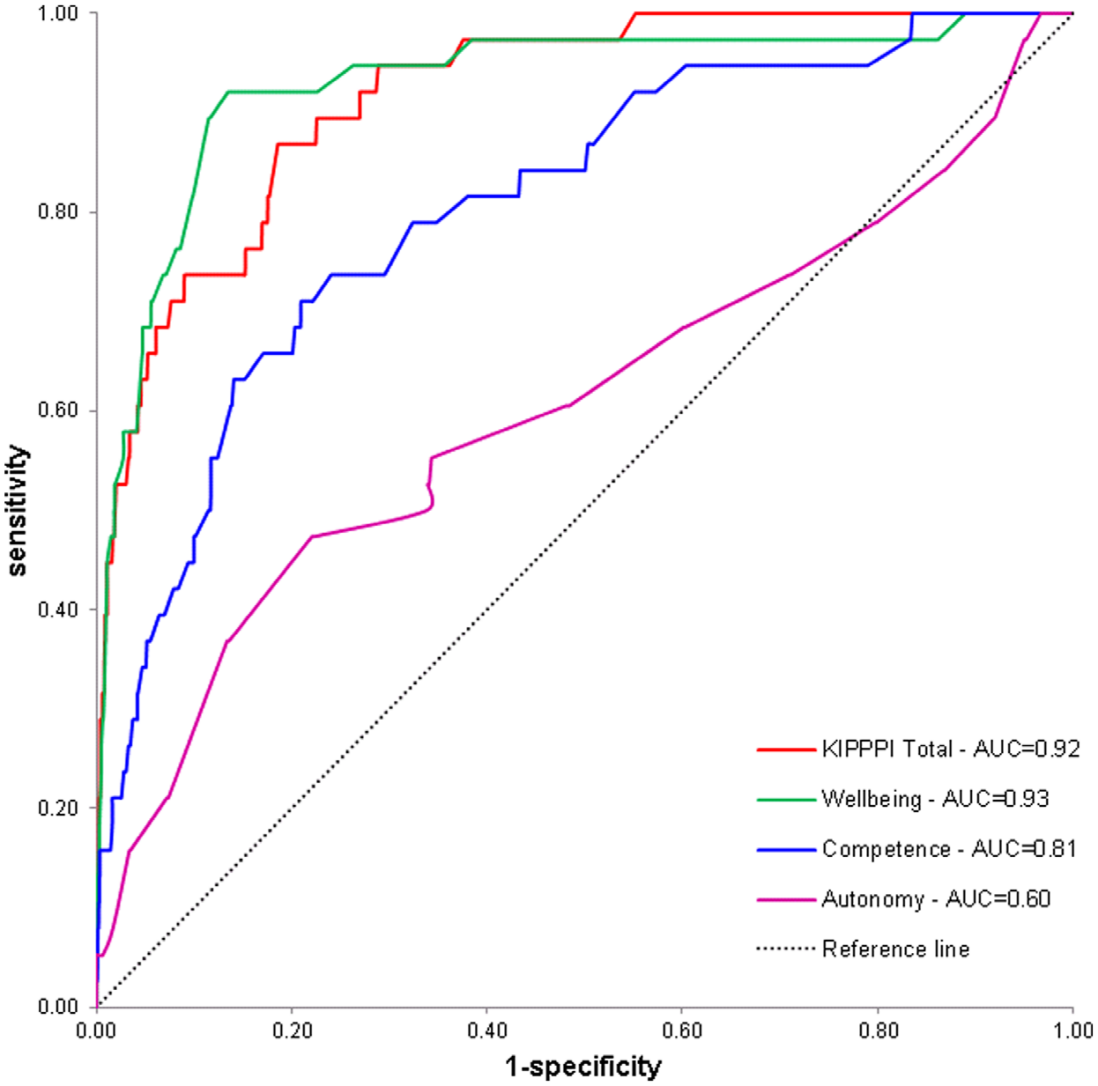
KIPPPI Receiver Operating Characteristic (ROC) curve. Receiver Operating Characteristic curve for KIPPPI scales Wellbeing, Competence, Autonomy and KIPPPI Total score, relative to CBCL1.5-5 Total Problem score in the clinical range. AUC = area under the curve.

We expected large AUCs for the KIPPPI total score and for the Wellbeing scale since the content of the items of these scales most resemble the items of the CBCL1.5-5 Total Problem score. Whereas, we expected the AUCs of the Competence scale and Autonomy scale to be small (i.e. closer to 0.50) since the content of items of the Competence scale and Autonomy scale are less reflected in the CBCL1.5-5 items.

**Table 6:** Area Under the Curve and sensitivity, specificity, liklihood ratio’s and Youden’s index for a range of KIPPPI scores relative to a clinical CBCL Total Problem score.

KIPPPI Total							KIPPPI Wellbeing							KIPPPI Competence							KIPPPI Autonomy						
AUC = 0.92 (95% CI = 0.88–0.96)							AUC = 0.93 (95% CI = 0.88–0.98)							AUC = 0.81 (95% CI = 0.74–0.88)							AUC = 0.60 (95% CI = 0.50–0.71)						
score	sens	spec	LHR+	LHR−	OR	J	score	sens	spec	LHR+	LHR−	OR	J	score	sens	spec	LHR+	LHR−	OR	J	score	sens	spec	LHR+	LHR−	OR	J
50	0.92	0.73	3.41	0.11	31.09	0.65	22	0.95	0.74	3.65	0.07	54.08	0.68	10	0.92	0.45	1.67	0.18	9.41	0.37	9	0.89	0.08	0.97	1.38	0.70	−0.02
51	0.89	0.76	3.71	0.14	25.62	0.65	23	0.92	0.78	4.18	0.10	40.77	0.70	11	0.87	0.50	1.74	0.26	6.69	0.37	10	0.84	0.13	0.97	1.23	0.78	−0.03
52	0.89	0.77	3.87	0.14	27.09	0.67	24	0.92	0.81	4.84	0.10	49.03	0.73	12	0.84	0.57	1.95	0.28	6.96	0.41	11	0.79	0.20	0.99	1.05	0.94	−0.01
53	0.87	0.80	4.35	0.16	26.77	0.67	25	0.92	0.84	5.75	0.10	60.38	0.76	13	0.82	0.62	2.16	0.29	7.43	0.44	12	0.74	0.29	1.04	0.90	1.16	0.03
**54**	**0.87**	**0.81**	**4.58**	**0.16**	**28.53**	**0.68**	**26**	**0.92**	**0.87**	**7.08**	**0.09**	**76.96**	**0.79**	14	0.79	0.68	2.47	0.31	7.99	0.47	13	0.68	0.40	1.13	0.80	1.42	0.08
55	0.82	0.82	4.56	0.22	20.75	0.64	27	0.89	0.88	7.42	0.13	59.33	0.78	15	0.74	0.72	2.64	0.36	7.32	0.46	14	0.61	0.52	1.27	0.75	1.69	0.12
56	0.76	0.85	5.07	0.28	17.94	0.61	28	0.82	0.90	8.20	0.20	41.00	0.72	16	0.74	0.76	3.08	0.34	9.01	0.50	15	0.55	0.66	1.62	0.68	2.37	0.21
57	0.74	0.87	5.69	0.30	19.05	0.61	29	0.76	0.92	9.50	0.26	36.42	0.68	**17**	**0.71**	**0.79**	**3.38**	**0.37**	**9.21**	**0.50**	**16**	**0.47**	**0.78**	**2.14**	**0.68**	**3.14**	**0.25**
58	0.74	0.89	6.73	0.29	23.03	0.62	30	0.74	0.93	10.57	0.28	37.81	0.67	18	0.66	0.83	3.88	0.41	9.48	0.49	17	0.37	0.87	2.85	0.72	3.93	0.23
59	0.74	0.89	6.73	0.29	23.03	0.63	31	0.71	0.94	11.83	0.31	38.36	0.65	19	0.63	0.86	4.50	0.43	10.46	0.49	18	0.21	0.93	3.00	0.85	3.53	0.14
60	0.74	0.90	7.40	0.29	25.62	0.64	32	0.68	0.95	13.60	0.34	40.38	0.64	20	0.55	0.88	4.58	0.51	8.96	0.44	19	0.16	0.97	5.33	0.87	6.16	0.12
61	0.74	0.91	8.22	0.29	28.78	0.65	33	0.58	0.96	14.50	0.44	33.14	0.54	21	0.47	0.90	4.70	0.59	7.98	0.37	20	0.08	0.98	4.00	0.94	4.26	0.06
62	0.71	0.92	8.88	0.32	28.16	0.63	34	0.58	0.97	19.33	0.43	44.65	0.55	22	0.42	0.92	5.25	0.63	8.33	0.34	21	0.05	0.99	5.00	0.96	5.21	0.05
63	0.68	0.93	9.71	0.34	28.23	0.61	35	0.58	0.97	19.33	0.43	44.65	0.55	23	0.39	0.94	6.50	0.65	10.02	0.33	22	0.05	1.00	.	0.95	.	0.05

Note: AUC = area under the curve; 95%CI = 95% confidence interval; sens = sensitivity; spec = specificity; LHR^+^ = liklihood ratio positive test; LHR^−^ = liklihood ratio negative test; OR = diagnostic odds ratio; J = Youden’s index. All AUC’s were significant (p<0.01). Scores with the highest unroundend Youden’s index are indicated in bold.

#### Clinical application

To evaluate the clinical application of the KIPPPI, registration data from the child health professionals (i.e. the clinical decision) was combined with the KIPPPI data from the parents.

The clinical application of the KIPPPI was explored by evaluating the relation between KIPPPI scores and the clinical decision of the child health professional. We hypothesised that the clinical decision of the child health professional to refer to another mental health professional or request a follow-up consultation, predicts higher KIPPPI scores, as high KIPPPI scores are expected to be indicative of problems. The data is hierarchical in nature since the child health professionals assessed more than one child, which makes (part of) the observations dependent on each other. Because the observations are not independent on each other, a multilevel regression analyses was used to evaluate the relation between the clinical decision as independent variable and the KIPPPI (scale-)scores as dependent variable, corrected for confounding effects of child’s gender and ethnicity.

**Table 7:** Clinical application of the KIPPPI; relation between KIPPPI scores and the decision by the child health professional to refer to a specialist and/or request a follow-up consultation.

	Referral or follow-up decision			
KIPPPI scales	Mean (SD)		Beta^1^	Effect size^2^
	Not referredN = 1335	ReferredN = 149		
KIPPPI TOTAL	41.8 (14.0)	53.0 (17.2)	11.00	0.65^b^
Physical health	1.0 (1.2)	1.3 (1.6)	0.33	0.19^c^
Wellbeing	16.1 (7.9)	21.3 (10.5)	4.95	0.50^b^
Eating/Drinking	2.2 (2.0)	2.7 (2.2)	0.57	0.23^c^
Sleeping	1.3 (1.4)	1.8 (1.7)	0.51	0.29^c^
Activity	3.4 (2.3)	4.2 (2.6)	0.77	0.31^c^
Mood	1.0 (1.3)	1.9 (2.2)	0.86	0.41^c^
Behaviour	8.4 (4.6)	11.4 (6.0)	3.21	0.50^b^
Competence	12.9 (7.3)	18.0 (8.4)	4.73	0.61^b^
Cognitive development	2.0 (1.5)	2.4 (1.7)	0.58	0.24^c^
Language	2.4 (2.6)	5.4 (3.6)	2.73	0.83^a^
Play	0.7 (1.0)	0.9 (1.3)	0.24	0.15^c^
Contact	7.5 (4.9)	9.2 (5.5)	1.69	0.31^c^
Autonomy	13.0 (3.4)	14.2 (3.7)	1.31	0.32^c^
Toilet training	8.0 (2.0)	8.7 (2.0)	0.68	0.35^c^
Motor skills	1.1 (0.8)	1.4 (1.0)	0.28	0.30^c^
Independence	4.0 (1.9)	4.5 (1.9)	0.43	0.26^c^

1 Unstandardized Beta’s are corrected for confounding effects of child’s gender and ethnicity and significant, *p*<0.05.

2 Difference of the means divided by SD in the subgroup ‘intervention needed’ and ‘referred’.

a indicates a large effect (*d*≥0.80).

b indicates a medium effect (0.50≤ *d* <0.80).

c indicates a small effect (0.20≤ *d* <0.50).

In this study we were able to combine 1448 (53.0%) of the parent-completed KIPPPI questionnaires with the clinical decision data registered by child health professionals. Combined data of 1284 (47.0%) children were lacking due to missing patient-codes. Significant differences (*p*<0.05) between the group with complete data and the group with incomplete data were found for the age of the child, ethnicity of the child and country of birth of the father and not for child gender, country of birth of the mother, age of the parents, family composition, person who completed the questionnaire and mean KIPPPI scores. Effect sizes of the significant differences between the group with complete data en the group with incomplete data, however, were very small (child age *d* = 0.16, ethnicity *d* = 0.05 and father country of birth *d* = 0.04) and indicate that the data may be interpreted as ‘missing at random’.

Multilevel regression analyses were performed in SAS software version 9.13 (SAS Institute Inc., 2009). All other analyses were performed in SPSS 19.0 (SPSS Inc. 2010).

## Results

### Score Distribution

Mean scale scores for the total sample and in subgroups by child’s gender and ethnic background are presented in Table 3. Compared to girls, boys had significantly (*p*<0.05) higher mean KIPPPI Total scores and higher mean scores on the scales Wellbeing, Competence and Autonomy, and on the subscales Activity, Behaviour, Language, Play, Toilet training, Motor skills and Independence. Compared to Native children, non-native children had significantly (*p*<0.05) higher mean KIPPPI Total scores and higher mean scores on the scales Wellbeing and Competence, and on the subscales Sleeping, Activity, Mood, Behaviour, Cognitive development, Language, Play, Contact and Independence. Non-native children had significantly (*p*<0.05) lower scores on the KIPPPI subscale Toilet training. See Table 3.

Floor effects were present for seven subscales: Physical health, Eating/Drinking, Sleeping, Mood, Language, Play and Motor Skills. Ceiling effects were absent (Table 3).

### Reliability

Internal consistency was 0.88 for the KIPPPI Total score; 0.86 for the Wellbeing scale; 0.83 for the Competence scale; and 0.58 for the Autonomy scale (Table 3). The internal consistency of the subscales is presented in Table 3. Only the subscales Eating/Drinking, Behaviour, Language and Contact had Cronbach alpha’s greater than 0.70.

Test-retest reliability was 0.80 for the KIPPPI Total score; 0.76 for the Wellbeing scale; 0.73 for the Competence scale; and 0.60 for the Autonomy scale (Table 3). The test-retest reliability of the subscales is presented in Table 3. Only the subscales Physical health, Sleeping, Activity, Behaviour, Language, Contact, Motor skills and Independence had ICCs greater than 0.70.

### Validity

#### Concurrent validity

As hypothesised, positive correlations were found between the KIPPPI Total score and the CBCL1.5-5 scores for Internalising (*r* = 0.60), Externalising (*r* = 0.63) and Total Problem score (*r* = 0.68). The KIPPPI Wellbeing scale was positively correlated with the CBCL1.5-5 scores for Internalising (*r* = 0.55), Externalising (*r* = 0.74) and Total Problem score (*r* = 0.72). The KIPPPI Competence scale was positively correlated with the CBCL1.5-5 scores for Internalising (*r* = 0.48), Externalising (*r* = 0.32) and Total Problem score (*r* = 0.43). The KIPPPI Autonomy scale was positively correlated with the CBCL1.5-5 scores for Internlising (*r* = 0.18), Externalising (*r* = 0.23) and Total Problem score (*r* = 0.23). All these correlations were significant, p<0.01. See Table 4 for the concurrent validity of the subscales.

#### Discriminative validity

KIPPPI scores of 2109 (77.2%) children of parents who did not report to be worried about their child’s upbringing were compared to KIPPPI scores of 604 (22.1%) children of parents who did report to be worried (percentages do not sum to 100% because of missing values). All regression coefficients were significant (p<0.01) and positive: KIPPPI Total, Β = 11.87; Wellbeing scale Β = 7.38; Competence scale, Β = 3.40; and Autonomy scale, Β = 1.10. See Table 5. The effect sizes of the differences in mean KIPPPI scores between parents that did and did not report to be worried about their child’s upbringing ranged from large to small: KIPPPI Total score, *d = *0.81; for the Wellbeing scale, *d = *0.85; for the Competence scale, *d* = 0.46; and for the Autonomy scale, *d* = 0.33. See Table 5 also for the effect sizes of the subscales. The subscales Activity and Behaviour had medium effect sizes whereas the effect size for all other subscales was small.

### Screening Accuracy

ROC curves of the KIPPPI Total score, Wellbeing scale, Competence scale and Autonomy scale are presented in Figure 1. In Table 6 AUC and sensitivity, specificity, LHR^+^, LHR^−^, OR and Youden’s index are presented for a range of KIPPPI cutpoints. The AUC for the KIPPPI Total score was 0.92 and for the Wellbeing scale 0.93. The AUC for the Competence and Autonomy scale were lower; respectively 0.81 and 0.60.

### Clinical Application

Child health professionals referred 149 (10.0%) children for further evaluation or requested a follow-up consultation. All regression coefficients were significant (*p*<0.05) and positive: KIPPPI Total score, Β = 11.00; Wellbeing scale, Β = 4.95; Competence scale, Β = 4.73; and Autonomy scale, Β = 1.31. The effect sizes of the differences in mean KIPPPI scores between children that did and did not need referral or a follow-up consultation were for the KIPPPI Total score *d* = 0.65; for the Wellbeing scale *d* = 0.50; for the Competence scale *d* = 0.61; and for the Autonomy scale *d* = 0.32. See Table 7 also for the effect sizes of the subscales. The subscale Language had a large effect size, the subscale Behaviour had a medium effect sizes and all other subscales had small effect sizes.

## Discussion

The present study evaluated the psychometric properties of the KIPPPI, a Dutch instrument that was developed to measure psychological and pedagogical problems in 2-year-olds, in a large community sample. The score distribution and the following psychometric properties of the KIPPPI were determined: internal consistency, test-retest reliability, concurrent validity, discriminative validity and screening accuracy. Additionally we also evaluated the clinical application of the KIPPPI.

### Score Distribution

The KIPPPI scales and KIPPPI Total score showed no floor or ceiling effects. Floor effects were present, however, for the following subscales: Physical health, Eating/Drinking, Sleeping, Mood, Language, Play and Motor Skills. This means that changes within toddlers with low scores for these subscales cannot be measured and that there is less differentiation possible between children with low KIPPPI scores (i.e. few psychosocial problems). [19] The mean KIPPPI Total score and KIPPPI scale scores were less favourable for boys compared to girls and for non-native children compared to native children. There was, however, no difference in mean score on the Autonomy scale between native and non-native children. These findings are in line with previous studies that report boys experience psychosocial problems more often than girls [26] and that psychosocial problems are more frequently reported in immigrant children compared to native children. [27], [28].

### Reliability

Internal consistency for the KIPPPI Total score, Wellbeing scale and Competence scale was adequate (>0.70) whereas the internal consistency for the Autonomy scale was marginal (i.e. 0.58). Lower internal consistency for the Autonomy scale might be explained by the inclusion of some items that assess behaviours that may not be expected to co-occur, for example: “Runs and climbs” and “Tries to repair something that is broken.”

The 5–78 day (*mean* = 38.6, *SD* = 17.5) test-retest reliability was adequate (>0.70) for the KIPPPI Total score, Wellbeing scale and Competence scale and was marginal (i.e. 0.60) for the Autonomy scale. These results mean that, assuming that no real changes in psychosocial problems occur, the KIPPPI Total score, Wellbeing scale and Competence scale provide stable outcome measures over time.

### Validity

As hypothesised, the KIPPPI showed good concurrent validity: the KIPPPI Total score and Wellbeing scale had large positive correlations with CBCL1.5-5 Internalising, Externalising and Total Problem scores. Also, as hypothesised the Competence scale and Autonomy scale had a small to medium positive correlation with CBCL1.5-5 Internalising, Externalising and Total Problem scores.

The KIPPPI Total score and scale scores were able to distinguish between parents who reported being worried about their child’s upbringing and parents who did not report being worried. This indicates that scores were less favourable for children of parents who were worried, compared to parents that were not worried. The difference between these subgroups in mean KIPPPI Total score and mean scores on the Wellbeing scale was large (*d*≥0.80) However, the difference in mean scores on the Competence scale and Autonomy scale was small (0.20≤ *d*<0.50). These results indicate good discriminative validity for the KIPPPI Total score and Wellbeing scale.

### Screening Accuracy

The KIPPPI showed large Areas Under the Curve (>0.90) for the KIPPPI Total score and Wellbeing scale and indicates that these scores have high accuracy in discriminating between children with psychosocial problems and children without psychosocial problems. The Competence scale AUC showed moderate accuracy (AUC = 0.81) and Autonomy scale AUC showed low accuracy (AUC = 0.60). The KIPPPI Total score and Wellbeing scale are better able to discriminate between children with psychosocial problems and children without psychosocial problems, compared to the Competence scale and Autonomy scale.

### Clinical Application

KIPPPI Total score and scale scores were positive and significantly associated with child health professional’s decision whether or not a follow-up consultation or referral was required. The difference between children who were referred and children we were not referred in mean KIPPPI Total score and mean scores on the Wellbeing scale and Competence scale was medium (0.50≤ *d*<0.80). However the difference between these subgroups in mean scores on the Autonomy scale was small (0.20≤d<0.50). These results indicate that scores were less favourable for children who were referred or asked back for a follow-up consultation, compared to children who were not referred or asked back for a follow-up consultation.

### Limitations and Strengths

Our study has two main limitations. First, in the current study we have no data on the non-response group, because no information is available on parents who did not attend the well-child visit. Therefore, some care should be taken with generalizing these results to the total population. However, due to the diversity of our large study population, we do not expect that the characteristics of the non-response group are very different of that from the study population. In the Netherlands, participation of parents with their child in the preventive youth health care is free of charge, which makes the well-child visit easily accessible for all population groups: There is no dissimilarity in visiting frequency between native Dutch and non-native children and their parents. [29].

Second, the report by parents introduces the proxy-problem: self-report by two-year-old children on their psychosocial problems is not possible, because children of this age lack the necessary language skills and the cognitive abilities to interpret the questions and they do not have a long-term view of events. [30] Although reports by parents do not provide first-hand information and answers might be clouded by how a parent interprets their child’s behaviour, proxy by parents in this case might be a useful alternative. [31].

A major strength of our study is the large and diverse sample. Additionally, the setting in which the respondents were invited to complete the KIPPPI, the daily practice of well-child-visit at the child health care centre, can be seen as either a strength or a limitation. We evaluated the psychometric properties in a setting in which the KIPPPI is used; however this specific setting might, on the other hand, hamper the generalizations of our results to other settings.

### Conclusions

The psychometric properties of the KIPPPI are comparable to that of other early detection tools for preschool children. [32] Early detection instruments for psychosocial problems in infants and toddlers are scarce. [33] The Child Behavioral Checklist (CBCL1.5-5) [16] has good reliability and validity, but is too long to employ as an early detection tool in preventive child health care. The KIPPPI addresses both problem behaviour as well as competencies, but unlike the Brief Infant-Toddler Social and Emotional Assessment (BITSEA) [34] and the Ages & Stages Questionnaire-Social-Emotional version (ASQ-SE) [35], the KIPPPI does not consist of items specifically for the early detection of autism spectrum disorders. The KIPPPI covers a wide range of psychological and pedagogical aspects of a child’s development, which might make it appealing to use by a child health professional during the well-child visit.

We recommend future studies to evaluate the psychometric properties of the KIPPPI, also in a different sample and setting. The setting of this study was the daily practice of a well-child visit in an urban area, however, it would be good to replicate this study in a more rural area, possibly outside the context of a well-child visit. Future studies may also wish to further investigate differences in KIPPPI psychometric properties for population subgroups (e.g. child gender and ethnic background). Furthermore, differences in screening accuracy and cutpoints for boys and girls might be explored, since these groups have different mean KIPPPI scores. Although the KIPPPI showed adequate screening accuracy relative to the CBCL1.5-5, we recommend further evaluation of the screening accuracy of the KIPPPI by including a clinical sample of children with a clinical diagnosis made by a (mental health) professional.

In conclusion, the results of our study support the reliability, validity and clinical application of the KIPPPI Total score, Wellbeing scale and Competence scale. Also, the screening accuracy of the KIPPPI Total score and Wellbeing scale were supported. The Autonomy scale needs further study.

## References

[1] American Academy of Pediatrics. Committee on children with disabilities: development surveillance and screening of infants and young children (2001) Pediatrics. 108: 192–196.

[2] American Academy of Pediatrics. Identifying infants and young children with development disorders in the medical home: an algorithm for developmental surveillance and screening (2006) Pediatrics. 118: 405–420. 10.1542/peds.2006-1231 16818591

[3] Hermans J, Ory F, Schrijvers G (2005) Supporting development and parenting: sooner, faster and better: an advice for early detection and interventions in regard to developmental and parenting problems. Julius Centrum: Utrecht.

[4] Klein Velderman M, Crone MR, Wiefferink CH, Reijneveld SA (2010) Identification and management of psychosocial problems among toddlers by preventive child health care professionals. Eur J Public Health 20: 332–338. 19858089 10.1093/eurpub/ckp169

[5] Reijneveld SA, Brugman E, Verhulst FC, Verloove-Vanhorick SP (2004) Identification and management of psychosocial problems among toddlers in Dutch preventive child health care. Arch Pediatr Adolesc Med 158: 811–817. 15289256 10.1001/archpedi.158.8.811

[6] Lavigne JV, Arend R, Rosenbaum D, Binns HJ, Christoffel KK, et al (1998) Psychiatric disorders with onset in the preschool years: I. Stability of diagnoses. J Am Acad Child Adolesc Psychiatry 37: 1246–1254. 9847496 10.1097/00004583-199812000-00007

[7] Mesman J, Koot HM (2001) Early preschool predictors of preadolescent internalizing and externalizing DSM-IV diagnoses. J Am Acad Child Adolesc Psychiatry 40: 1029–1036. 11556626 10.1097/00004583-200109000-00011

[8] Durlak JA, Wells AM (1998) Evaluation of indicated preventive intervention (secondary prevention) mental health programs for children and adolescents. Am J Community Psychol 26: 775–802. 9861693 10.1023/a:1022162015815

[9] Elliot J, Prior M, Merrigan C, Ballinger K (2002) Evaluation of a community intervention programme for preschool behaviour problems. J Paediatr Child Health 38: 41–50. 11869399 10.1046/j.1440-1754.2002.00713.x

[10] Verhulst FC, van der Ende J (1997) Factors associated with child mental health service use in the community. J Am Acad Child Adolesc Psychiatry 36: 901–909. 9204667 10.1097/00004583-199707000-00011

[11] Brugman E, Reijneveld SA, Verhulst FC, Verloove-Vanhorick SP (2001) Identification and management of psychosocial problems by preventive child health care. Arch Pediatr Adolesc Med 155: 462–469. 11296073 10.1001/archpedi.155.4.462

[12] Glascoe FP (2005) Screening for developmental and behavioral problems. Ment Retard Dev Disabil Res Rev 11: 173–179. 16161092 10.1002/mrdd.20068

[13] Sand N, Silverstein M, Glascoe FP, Gupta VB, Tonniges TP, et al (2005) Pediatricians’ reported practices regarding developmental screening: Do guidelines work? Do they help? Pediatrics 116: 174–179. 15995049 10.1542/peds.2004-1809

[14] Sturner RA (1991) Parent questionnaires - Basic office equipment. J Dev Behav Pediatr 12: 51–54. 2016403

[15] Kousemaker NPJ (1996) Zoeken, vinden, zorgen delen: de ontwikkeling van een praktijkparadigma voor onderkenning en pedagogische preventie van psychosociale problematiek in de Jeugdgezondheidszorg [Searching, finding, sharing care; the development of a practise paradigm for the descernment and pedagogical prevention of psychosocial problems in Youth Healthcare].

[16] Achenbach TM, Rescorla LA (2000) Manual for the ASEBA Preschool Forms & Profiles. Burlington: VT: University of Vermont, Research Center for Children, Youth, and Families.

[17] Definitions ‘native’ and ‘immigrant’. Statistics Netherlands website. Available: http://www.cbs.nl/nl-NL/menu/methoden/begrippen/default.htm?conceptid=37. Accessed 2012 Oct 18.

[18] Shrive FM, Stuart H, Quan H, Ghali WA (2006) Dealing with missing data in a multi-question depression scale: a comparison of imputation methods. BMC Med Res Methodol 6: 57. 17166270 10.1186/1471-2288-6-57 PMC1716168

[19] Terwee CB, Bot SDM, de Boer MR, van der Windt D, Knol DL, et al (2007) Quality criteria were proposed for measurement properties of health status questionnaires. J Clin Epidemiol 60: 34–42. 17161752 10.1016/j.jclinepi.2006.03.012

[20] Nunnally JC, Bernstein IH (1994) Psychometric theory. New York: McGraw-Hill.

[21] Cohen J (1988) Statistical power analysis for the behavioral sciences (2nd ed.). Hillsdale, NJ: Lawrence Erblaum Associates.

[22] Glascoe FP (1997) Parents’ concerns about children’s development: Prescreening technique or screening test? Pediatrics 99: 522–528. 9093291 10.1542/peds.99.4.522

[23] Swets JA (1988) Measuring the accuracy of diagnostic systems. Science 240: 1285–1293. 3287615 10.1126/science.3287615

[24] Fischer JE, Bachmann LM, Jaeschke R (2003) A readers’ guide to the interpretation of diagnostic test properties: clinical example of sepsis. Intensive Care Med 29: 1043–1051. 12734652 10.1007/s00134-003-1761-8

[25] Glas AS, Lijmer JG, Prins MH, Bonsel GJ, Bossuyt PMM (2003) The diagnostic odds ratio: a single indicator of test performance. J Clin Epidemiol 56: 1129–1135. 14615004 10.1016/s0895-4356(03)00177-x

[26] Kraemer S (2000) The fragile male. Br Med J 321: 1609–1612. 11124200 10.1136/bmj.321.7276.1609 PMC1119278

[27] Reijneveld S, Harland P, Brugman E, Verhulst F, Verloove-Vanhorick S (2005) Psychosocial problems among immigrant and non-immigrant children - Ethnicity plays a role in their occurrence and identification. Eur Child Adolesc Psychiatry 14: 145–152. 15959660 10.1007/s00787-005-0454-y

[28] Vollebergh WAM, ten Have M, Dekovic M, Oosterwegel A, Pels T, et al (2005) Mental health in immigrant children in the Netherlands. Soc Psychiatry Psychiatr Epidemiol 40: 489–496. 16003599 10.1007/s00127-005-0906-1

[29] Parents positive about child health clinics. Statistics Netherlands website. Available: http://www.cbs.nl/nl-NL/menu/themas/vrije-tijd-cultuur/publicaties/artikelen/archief/2005/2005-1734-wm.htm. Accessed 2012 Oct 18.

[30] Vogels T, Verrips GHW, Verloove-Vanhorick SP, Fekkes M, Kamphuis RP, et al (1998) Measuring health-related quality of life in children: the development of the TACQOL parent form. Qual Life Res 7: 457–465. 9691725 10.1023/a:1008848218806

[31] Theunissen NCM, Vogels TGC, Koopman HM, Verrips GHW, Zwinderman KAH, et al (1998) The proxy problem: child report versus parent report in health-related quality of life research. Qual Life Res 7: 387–397. 9691719 10.1023/a:1008801802877

[32] Sosna T, Mastergeorge A (2005) Compedium of screening tools for early childhood social-emotional development. Available: http://www.cimh.org/downloads/The%20Infant, %20Preschool,%20Family, %20Mental%20Health%20Initiative%20Compendium%20of%20Screening%20Tools%20for%20Early%20Childhood%20Social-Emotional%20Deve.pdf. Accessed 2012 Oct 18.

[33] Glascoe FP (2000) Early detection of developmental and behavioral problems. Pediatr Rev 21: 272–280. 10922024 10.1542/pir.21-8-272

[34] Briggs-Gowan MJ, Carter AS, Irwin JR, Wachtel K, Cicchetti DV (2004) The brief infant-toddler social and emotional assessment: Screening for social-emotional problems and delays in competence. J Pediatr Psychol 29: 143–155. 15096535 10.1093/jpepsy/jsh017

[35] Squires J (2002) Ages & Stages Questionnaire Social-Emotional: A parent-completed, child monitoring system for social-emotional behaviors. Baltimore: Paul H. Brookes Publishing Co., Inc.

